# A Study of Crystalline Mechanism of Penetration Sealer Materials

**DOI:** 10.3390/ma7010399

**Published:** 2014-01-14

**Authors:** Li-Wei Teng, Ran Huang, Jie Chen, An Cheng, Hui-Mi Hsu

**Affiliations:** 1 Department of Harbor and River Engineering, National Taiwan Ocean University, No. 2 Pei-Ning Road, Keelung 20224, Taiwan; E-Mails: dlwkimo@gmail.com (L.-W.T.); ranhuang@ntou.edu.tw (R.H.); 2 Department of Civil Engineering, National Ilan University, No. 1, Sec. 1, Shen-Lung Road, I-Lan 26047, Taiwan; E-Mails: jchen@niu.edu.tw (J.C.); ancheng@niu.edu.tw (A.C.)

**Keywords:** crystalline penetration sealer materials (CPSM), waterproof, C-S-H gel, water permeability, rapid chloride permeability test (RCPT), mercury intrusion porosimetry (MIP), X-ray diffraction (XRD), scanning electron microscope (SEM), fourier transform infrared spectroscopy (FT-IR), thermogravimetric analyzer (TGA)

## Abstract

It is quite common to dispense a topping material like crystalline penetration sealer materials (CPSM) onto the surface of a plastic substance such as concrete to extend its service life span by surface protections from outside breakthrough. The CPSM can penetrate into the existing pores or possible cracks in such a way that it may form crystals to block the potential paths which provide breakthrough for any unknown materials. This study investigated the crystalline mechanism formed in the part of concrete penetrated by the CPSM. We analyzed the chemical composites, in order to identify the mechanism of CPSM and to evaluate the penetrated depth. As shown in the results, SEM observes the acicular-structured crystals filling capillary pores for mortar substrate of the internal microstructure beneath the concrete surface; meanwhile, XRD and FT-IR showed the main hydration products of CPSM to be C-S-H gel and CaCO_3_. Besides, MIP also shows CPSM with the ability to clog capillary pores of mortar substrate; thus, it reduces porosity, and appears to benefit in sealing pores or cracks. The depth of CPSM penetration capability indicated by TGA shows 0–10 mm of sealer layer beneath the concrete surface.

## 1. Technological Approaches for Improving Concrete Durability

Concrete’s high versatility makes it an ideal tool for building projects of all shapes and sizes. Nowadays, rapid chloride (RC) construction is developing in full swing, because it is necessary to expose the concrete’s structures to harsh natural environments. The internal pore space of the RC structures will allow water, acidic and alkaline pollutants to infiltrate into the concrete and cause corrosion of reinforcing steel bars, and concrete cracks as a result of less service life [1,2]. In order to prevent erosion and destruction of pollutants, proprietors have attached increasing importance to its waterproofing for considering the common deficiencies of RC structures including cracks, peeling, spalling, honeycombs, water leakage, exposure of reinforcement bar, corrosion of steel bars, insufficient bearing capacity, *etc*. The fundamental cause of these deficiencies is insufficient durability of material, namely the RC degradation which includes two aspects: degradation of concrete and corrosion of reinforcing steel bars [3]. Degradation of concrete mainly includes carbonization of concrete (neutralization), dissolutive corrosion, alkali aggregate reaction, corrosion by erosive agents, freeze thawing damage, and concrete crack. Not only will concrete degradation reduce performance of concrete, but it will also accelerate the corrosion of reinforcing steel bars in the concrete. The occurrence of durability issues such as freeze thawing, carbonization, corrosion and alkali aggregate reaction and concrete cracks are inextricably linked to three factors, namely: porosity of concrete, alkali content of concrete and presence of aqueous medium. Hence, how to reduce the porosity and alkali content of concrete, and to come off effective concrete waterproof protection, has become a key issue to ensure concrete durability.

The common practice is to reinforce and repair the concrete with organic polymer materials when there are major damages [4]. However, under these circumstances (concrete porosity, alkali content of concrete and presence of aqueous medium) hidden danger cannot be removed completely, because organic polymer material can only protect the surface of concrete without any changes to both of the key factors: porosity and alkali content. At present, the crystalline penetration sealer materials (CPSM) provide a fundamental solution to concrete durability and they have been applied and popularized in a large number of projects [5–7].

The CPSM features good hydrophilicity, which allows it to penetrate deeply into the concrete. The alkali content in the interior of conventional concrete is much more than that in the surface area. The CPSM can penetrate into the interior of concrete and chemically react with alkali with the presence of water and generate silicone gel, covering the capillary channels. The layer of silicone gel once hydrated will become a solid and acicular-structured substance and fill all capillary channels beneath the concrete surface [8], with its resultants being a permanent part of the concrete. Its penetration will reduce the alkali content within the concrete and prevent intrusion of external harmful substances [9]. If water is present, this process will keep repeating until the concrete is completely sealed up.

Even though all kinds of test data and research have praised CPSM’s advantages in both theoretical technologies and application functions, which are accepted as an agreed fact, the major components and those proportions have not been fully announced in public or research [10]. It is the authors’ intention to introduce a kind of CPSM to enhance concrete usage life span and durability similar to or even better than the ones available in market. Therefore, we performed a complete product evaluation to gain knowledge of a product for those of commercial CPSM. A similar product of CPSM had been proposed in a previous article [11]. As discussed in this article, the laboratory set up a test plan to perform a series of experimental tests on samples to determine if they contain crystalline penetration, what kind of crystals those are, whether or not they are capable of sealing pores or cracks and, finally, the depth of crystalline penetrations.

## 2. Experiments and Discussions

### 2.1. Crystalline Penetration Sealer Materials (CPSM)

**Figure 1.**
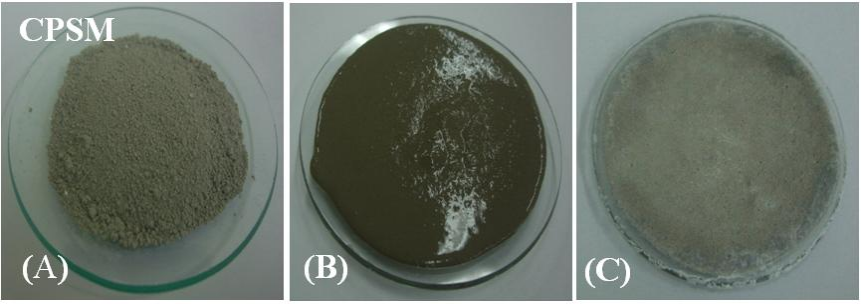
The photos of before, during and after hydration process for the same sample. (**A**) before hydration; (**B**) during hydration; (**C**) after hydration.

The CPSM, an inorganic material of grey color, is a man-made material of powered coating waterproof which primarily constitutes ingredients as cement, silica sand, and chemical compounds, by mixing together those ingredients as a form of gel which develops strength by chemical reaction with water by formation of hydrate. The gel once hydrated will possibly become a solid, ventilate and acicular-structured substance and fill all capillary channels beneath the concrete surface, with its resultants being a permanent part of the concrete. The photos of before, during and after hydration show the formation of hydrate for water/CPSM ratio 2/5 by weight as in Figure 1A–C, wherein Figure 1A,B indicate without and with water; Figure 1C shows the white crystals appear at the top of the sample after 24 h of hydration. It is necessary to point out that the previous study [11] had investigated two commercial products of CPSM and then proposed a list of components for one kind of similar CPSM, which are the same components used here to produce all of these CPSM test samples.

### 2.2. Test Samples

There are two kinds of concrete test specimens, with and without CPSM (CPSM coated and uncoated) which have the same concrete mix design as indicated in Table 1, which were prepared for the following testing. The main samples were treated by two layers of CPSM topping and the control samples were left untreated. The concrete test specimens were produced at the standard curing age of concrete of 28 days, in which moist cured for 7 days, involving maintenance conditions of desired moisture 95% and temperature 20 ± 2 °C, followed by 21 days air cured of 70% and 24 ± 6 °C, respectively.

### 2.3. Water Permeability Test and Rapid Chloride Permeability Test (RCPT)

To see if CPSM may form an effective screen by generating silicone gel within the concrete which seals the capillary channels and micro cracks, we first remove the CPSM topping from the main samples and then perform two permeability tests on both the main and control samples, namely the CRD-C48-92 standard test method for water permeability of concrete and ASTM C1202-12 RCPT [12]. In other words, we used a permeability method intended to compare the CPSM coated and uncoated groups with each other.

**Figure 2.**
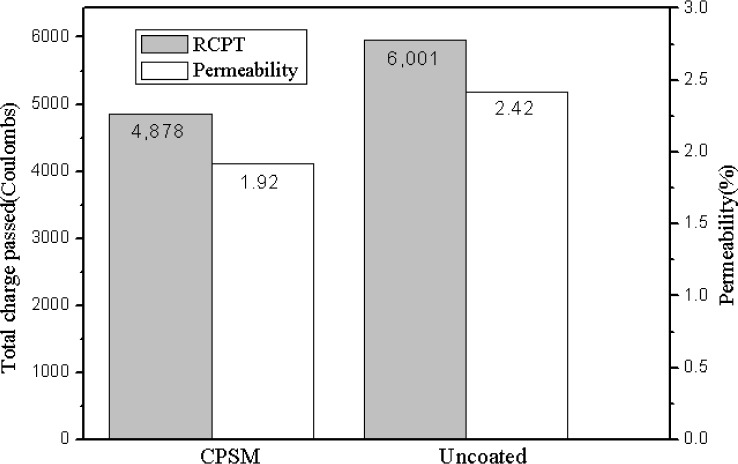
Water permeability test and rapid chloride permeability test (RCPT).

The laboratory testing of these specimens reports a similar trend for the CPSM coating group, as indicated in Figure 2, which is a histogram that indicates the amount of both types of permeability with each condition, recording 20.62% of less water permeability and 18.71% of lower current measured when estimating the resistance of the specimen to chloride ion penetration. In other words, the CPSM treated samples show less water penetration through samples in comparison to the control samples.

The corrosion of chloride is one of the key factors that deteriorate concrete. Once free Cl^−^ in the environment penetrates into the concrete, diffluent CaCl_2_ and a large amount of crystal water as well as solid compound with a several-fold larger size will be generated, accelerating the corrosion of reinforcing steel bars and causing expansion and cracking of concrete basal body. However, if the CPSM with water as its carrier penetrates into the interior of the concrete and chemically reacts with free alkali and generates silicone gel which seals the capillary channels and micro cracks within the concrete, the samples should exhibit good resistance to both chloride and water. This clearly explains why the CPSM coating samples possess less impermeability.

### 2.4. Mercury Intrusion Porosimetry (MIP)

The ASTM D4404-10 [13] standard test method takes the mercury intrusion porosimetry method (MIP) in determining the volume and the volume distribution of pores in soil and rock with respect to the apparent diameter of the entrances of the pores. In general, both the size and volume of the pores affect the performance of soil and rock. Thus, the pore volume distribution is useful in understanding soil and rock performance and in identifying a material that can be expected to perform in a particular manner, particularly CPSM in this case. The hydrated cement is a highly porous material with a continuous range of pore sizes from nanometers to micrometers. The pores, which are contained within the calcium silicate hydrate phase of the cement paste (C-S-H gel) and are the remnants of space previously occupied by mixing water (referred to as capillary pores), are characterized as C-S-H gel pores and capillary pores depending on the pore sizes found in MIP. The range of apparent diameters of pores for this test method is applicable by the operating pressure range of the testing instrument. This range is typically between apparent pore entrance diameters of about 100,000 nm (100 μm) and 2.5 nm (0.0025 μm), in which 10 nm is the line drawn between the C-S-H gel pores and capillary pores [14].

**Figure 3.**
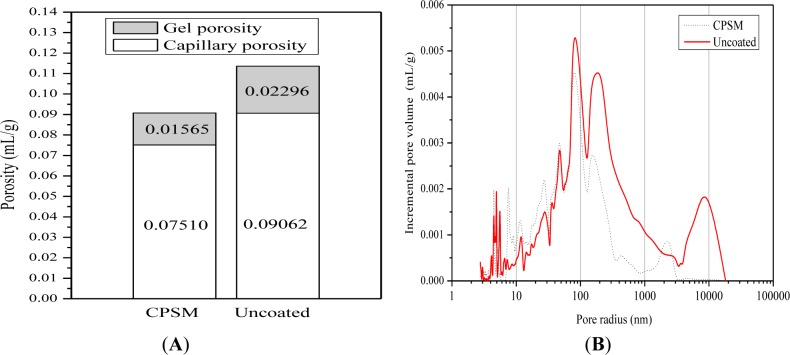
Mercury intrusion porosimetry (MIP), the volume and the volume distribution of pores. (**A**) volume change of pores; (**B**) volume distribution of pores.

As shown in Figure 3A, both values of porosity are smaller for the CPSM coating samples, giving a total sum of nearly 20%. This may explain the observation that the CPSM with water as its carrier penetrates into the interior of the concrete and chemically reacts with free alkali and generates silicone gel which partially seals the capillary channels and micro cracks within the concrete; thus, the penetration reduces the CPSM coating samples’ porosity as a return. This situation leads to a different pore volume distribution and the distinction between the two different classes of pores, mostly between 100 nm and 10,000 nm (see Figure 3B, 100–10,000 nm), which is in the range of capillary pores or represents more reduced capillary pores instead of the C-S-H gel pores due to the penetration.

### 2.5. Sieve Analysis

**Figure 4.**
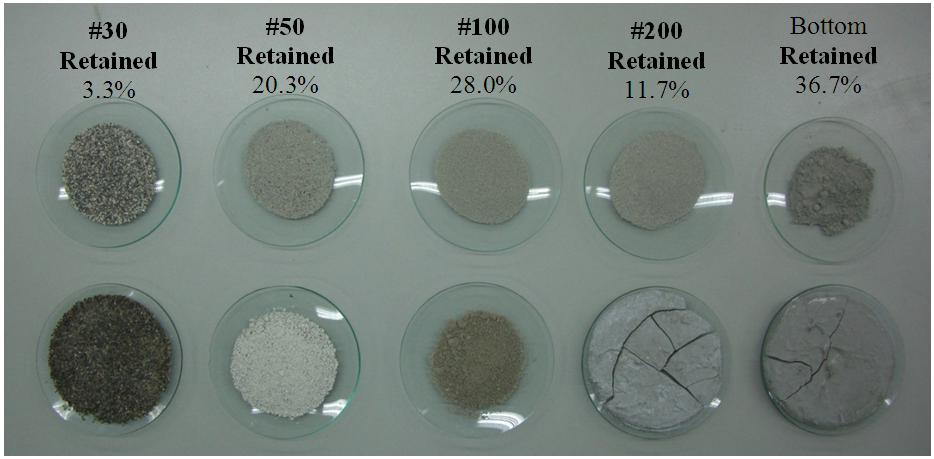
Five discrete particle size fractions and their mixes with water.

A sieve analysis involving a nested column of sieves with wire mesh cloth was performed on a CPSM aggregate sample. A suitable sieve size for the sample was selected and placed in order of decreasing size, from top to bottom, in a mechanical sieve shaker. A pan was placed underneath the nest of sieves to collect the aggregate that passes through the smallest. After the aggregate reaches the pan, the amount of material retained in each sieve is then weighed as five discrete particle size fractions ranging from sieves No. 30, No. 50, No. 100, No. 200+ (down to the pan) being extracted in weight percentages indicated in Figure 4. The distribution of cement particles is in the range of 0.001–0.1 mm [15], which is close to mesh size No. 200+ (75 μm); thus, this may indicate that almost a half of the total weight is contributed by the cement. Meanwhile, the particle distribution of silica sands is in the range of 0.015–0.23 mm, namely No. 100 (150 μm), and it gives about 28% of total weight. Regarding the remaining 24% of total weight for the substances retained in sieves No. 30 and No. 50, it may require more laboratory works to determine their ingredients.

We further observed the role of these constituents by adding water to each of those fractions, from which only the fractions of No. 200+ became increasingly hard with time. This observation further verifies the possible materials retained in different sieves, all of which can be classified as two different groups, namely No. 200+, the cement-like group, and the rest making up a silica sand-like group. This also explains why a possible cement hydration was observed in the former. In addition, in No. 50, we observed white compounds, which are the same materials as found in Figure 1C or the white crystals that appeared at the top of CPSM test samples after 24 h of hydration. These white materials obviously differ from silica sands; thus, No. 50 may be key to exploring CPSM for its solid substances at all capillary channels beneath the concrete surface. Details of exploration of these constituents will be discussed later.

### 2.6. X-Ray Diffraction (XRD)

#### (1) Various depths of penetration of substances

**Figure 5.**
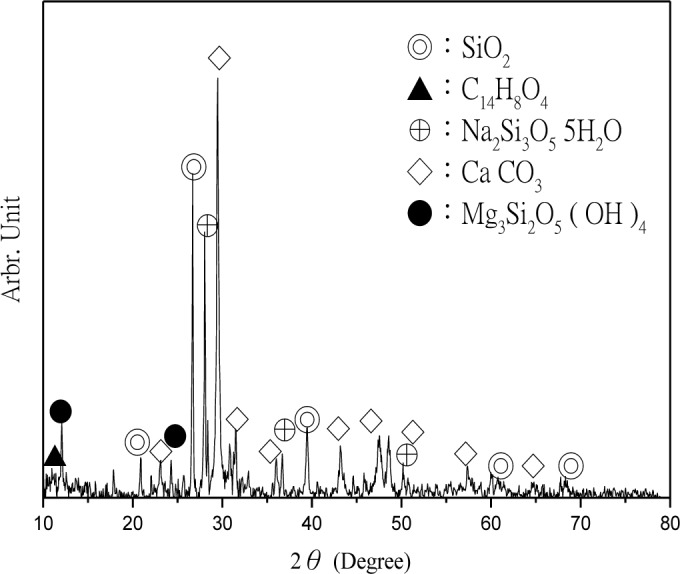
XRD of crystalline penetration sealer materials (CPSM)’s topping.

**Figure 6.**
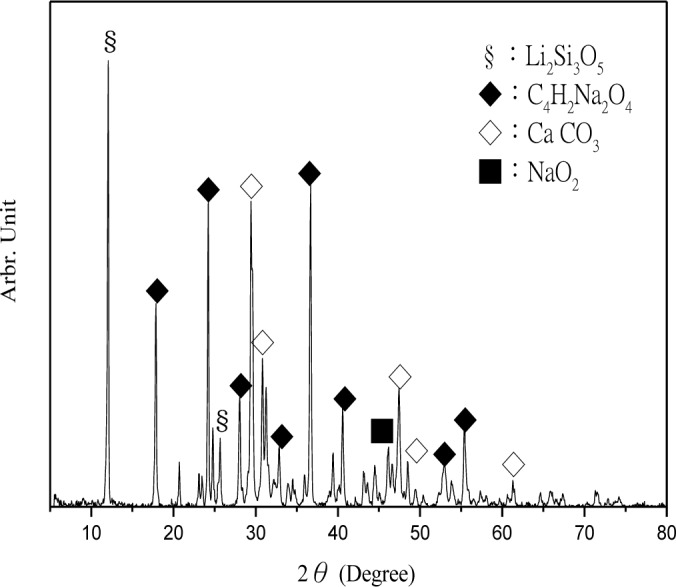
XRD of acicular-structured substance.

**Figure 7.**
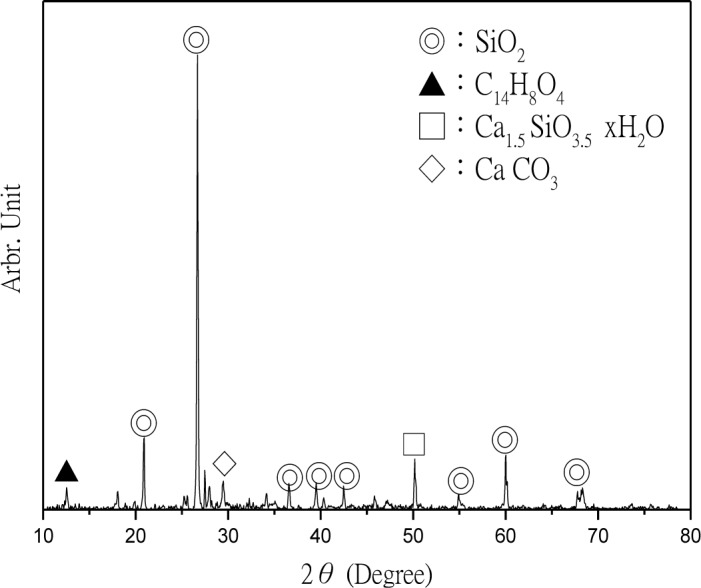
XRD of 5 mm beneath the surface.

**Figure 8.**
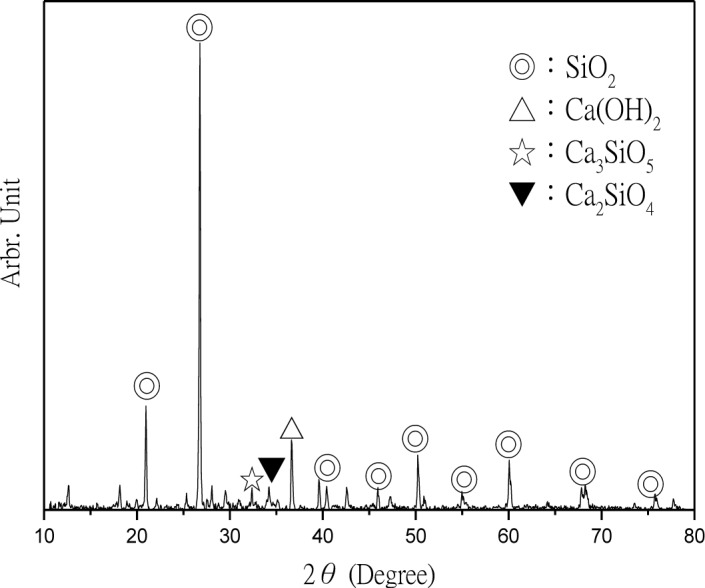
XRD of 20 mm beneath the surface.

The CPSM can penetrate into the interior of concrete and chemically react with alkali in the presence of water to generate silicone gel and then cover the capillary channels. The layer of silicone gel once hydrated will become a solid and acicular-structured substance and fill all capillary channels beneath the concrete surface. The deeper it penetrates, the more it will reduce the alkali content within the concrete and prevent intrusion of external harmful substances. Thus, XRD analyses were performed to investigate CPSM’s topping (Figure 5), acicular-structured substance (Figure 6), and two layers of substance at 5 and 20 mm beneath the topping (Figures 7 and 8), respectively.

XRD analysis identified the main components of the topping CPSM as SiO_2_, C_14_H_8_O_4_, Na_2_Si_3_O_5_·5H_2_O, CaCO_3_, and Mg_3_Si_2_O_5_, as indicated in Figure 5. The main components of the acicular-structured substance are Li_2_Si_3_O_5_, C_4_H_2_Na_2_O_4_, CaCO_3_ and NaO_2_. To a depth of 5 mm beneath the topping, the penetrated CPSM reacts with Ca(OH)_2_ to form Ca_1.5_SiO_3.5_·*x*H_2_O and CaCO_3_, and it also explains the lack of Ca(OH)_2_ as shown in Figure 7. To a depth of 20 mm beneath the surface, we observe the compounds SiO_2_, Ca(OH)_2_, Ca_3_SiO_5_ and Ca_2_SiO_4_ due to cement hydration. This clearly identifies the nonexistence of CPSM penetration at that depth.

#### (2) Various sieves of substances mixing with water

**Figure 9.**
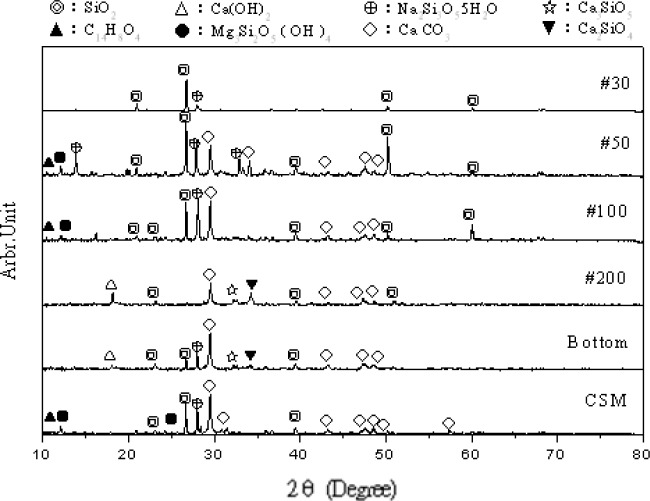
XRD of Various sieves of substances mixing with water.

Taking the sieve analysis results and the corresponding hydrates as indicated in Figure 4, XRD analyses were performed to investigate their components as shown in Figure 9. For the No. 30, the major component is SiO_2_. For the No. 50, the white compounds majorly comprise CaCO_3_, Na_2_Si_3_O_5_·5H_2_O, and SiO_2_, in which the last two have diffraction intensity differences respectively for peaks at 32.9°2θ and 34.1°2θ in comparison with other sieves, indicating that these two compounds may be key to the crystalline structures. For No. 100, the major component is SiO_2_, which means a close diffraction pattern to that of No. 30. For No. 200+, diffraction intensity peaks at 32.3°2θ and 34.2°2θ indicate the elements of cement clinker, *i.e.*, Ca_3_SiO_5_, Ca_2_SiO_4_ and Ca(OH)_2_ (traditionally called slaked lime).

### 2.7. Scanning Electron Microscope (SEM)

**Figure 10.**
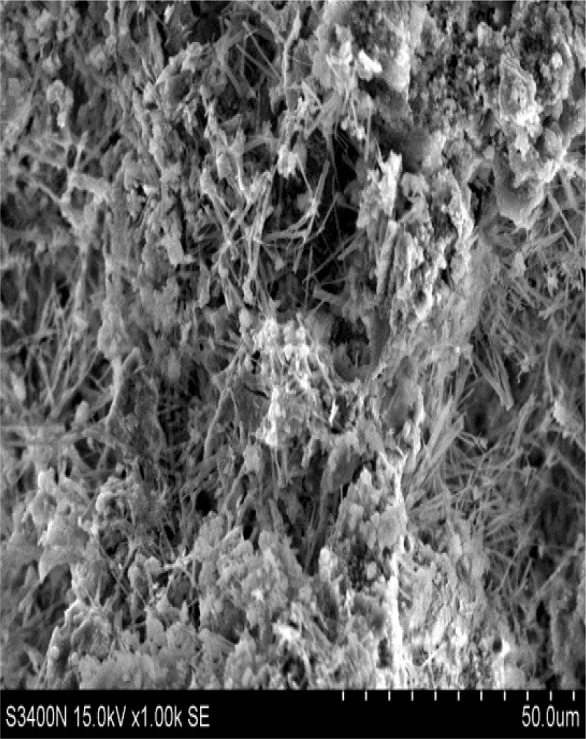
SEM (1000×) 5 mm beneath the surface.

To see an interaction between CPSM and substrate, SEM photographs (1000× magnification) of the substrate sample were used to give a visualized observation. Figure 10 illustrates the sample surface of cross-sections at 5 mm beneath the surface observed under SEM after a CPSM penetration, which shows that an indefinite number of acicular-like crystals were substantially and uniformly distributed to the surface of the substrate. It can be explained by showing that CPSM reacts with the alkaline substance of the concrete and generates silicone gel which imbeds the capillary channels of concrete structure and forms an indivisible screening layer. As a result the concrete surface density is enhanced. Moreover the intrinsic rigidity of silicon crystal strengthens the concrete structure and contributes the concrete surface strength. Hence, CPSM facilitates the enhancement of concrete surface strength; compressive strength tests had been performed to verify the enhancement.

**Figure 11.**
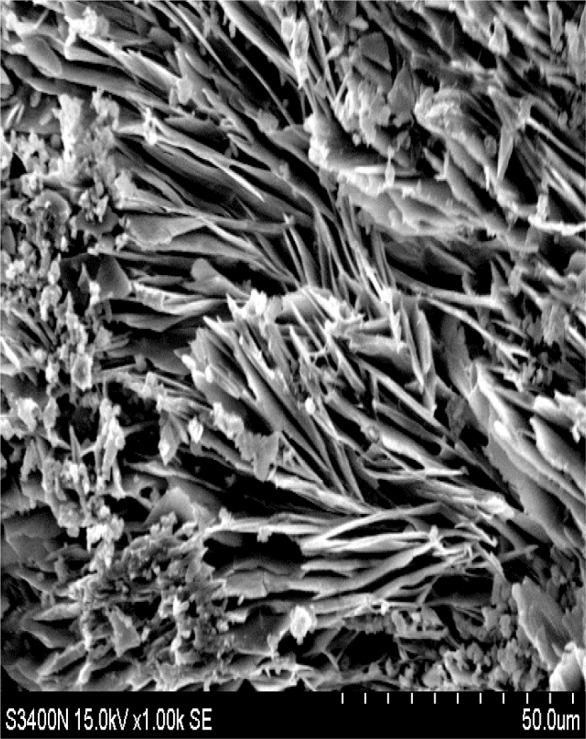
SEM (1000×) No. 50 CPSM.

In addition, the previous study [11] reveals high similarity between CPSM and cement; hence, the CPSM samples should be kept moist for at least seven days while curing to develop those acicular-like crystals to achieve their maximum intrinsic rigidity. Furthermore, Figure 11 illustrates the partial CPSM (No. 50) observed under SEM after mixing with water, which shows a large number of acicular-structured crystals, namely the white compounds shown in Figure 4. This observation clearly verifies that the No. 50 component of CPSM is the key substance to forming those penetrating crystalline materials.

### 2.8. Fourier Transform Infrared Spectroscopy (FT-IR)

**Figure 12.**
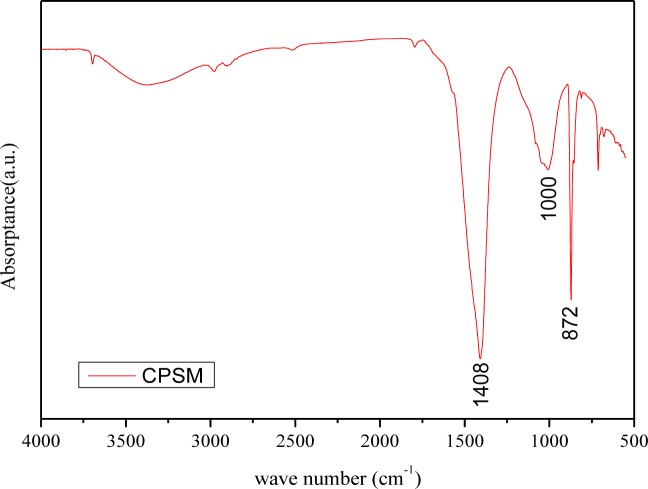
FT-IR spectrum.

Fourier transform spectroscopy is a measurement technique whereby spectra are collected based on measurements of the coherence of a radiative source. A Fourier transform is required to convert the raw data into an actual spectrum to infer how much light there is at each wavelength. FT-IR is a technique used to identify various functional groups in unknown substances through the identification of different covalent bonds that are present in the compound. For the infrared spectrum originating from the CPSM penetrated sample, most IR frequency absorptions are under 1500 cm^−1^, representing inorganic compounds, as expected for the test sample. There are three possible peak assignments for experimental absorption frequencies of 1410 cm^−1^, 984 cm^−1^, and 873 cm^−1^, respectively. By comparing the absorptions seen in the experimental spectrum to the literature regarding absorptions of various functional groups [16–18], we can determine a list of possible identities for the bonds present, *i.e.*, the ionic Si-O bond is a major one. For this type of vibration, an absorption range frequency displaying 1080–970 cm^−1^ indicates most likely C-S-H gel, while 872 cm^−1^ or 1410 cm^−1^ may represent CaCO_3_ (See Figure 12). Therefore, it can be concluded that, during the hydration period of a mix of CPSM and water, the compound silicon oxygen4 [SiO_4_^−^] develops the Si-O bond to form the siloxane linkage (Si-O-Si), the most abundant chemical bond in the earth’s crust, finally resulting in C-S-H gel and CaCO_3_.

### 2.9. Thermogravimetric Analysis (TGA)

The thermogravimetric technique has been applied to the examination of water of crystallization and comparison of varnishes and other surface coatings. A common application of TGA is to determine inorganic (e.g., crystal) content in a sample, which may be useful for corroborating predicted material structures. The TGA result is usually reported in the form of curves relating the mass lost from the sample against temperature. In this form, the temperatures at which certain processes begin and are completed are graphically demonstrated. The TGA curves obtained from heating different depths of the CPSM penetrated samples from 30 °C to 1000 °C are shown in Figure 12 and tabulated in Table 2. The curves show the loss in weight that occurred at different temperatures, as different types of weight (water) loss. EI-Jazairi and Illston [19] used the TG-DTG curve and semi-isothermal curve to study cement hydrates that were subject to temperature variation. The similarity between CPSM and cement is that both result in C-S-H gel and CaCO_3_ as previously mentioned. Hence, their study was used as follows to investigate crystalline formation at different penetration depths for the CPSM penetrated samples.

**Figure 13.**
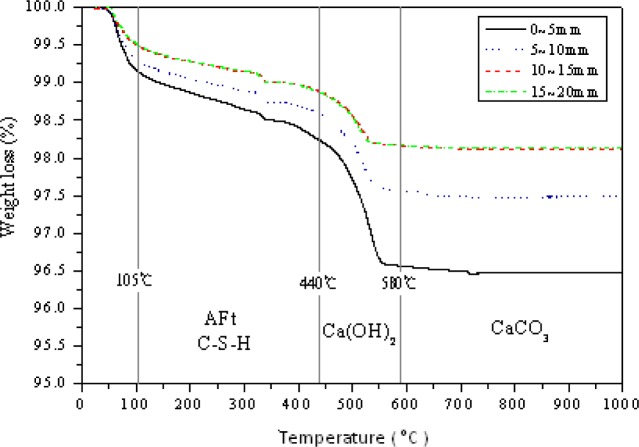
Thermogravimetric analyzer (TGA) curve.

From Table 2 and Figure 13, it can be observed that between 105 °C and 440 °C, the hydration reaction is manly that of AFt and C-S-H gel. Their weight losses are quite obvious for CPSM (0–5 mm) and CPSM (5–10 mm) but not for CPSM (10–15 mm) and CPSM (15–20 mm). Thus, it can be said that the CPSM may effectively penetrate into the depth of 0–10 mm. Meanwhile, between 440 °C and 580 °C, the hydration reaction is manly that of Ca(OH)_2_, which travels through capillary channels to the substrate to instigate its crystalline formation at a depth of 0–10 mm. As a final stage after 580 °C, hydration reaction is manly that of CaCO_3_; it still remains a general tendency in the larger weight loss for CPSM (0–5 mm) and CPSM (5–10 mm). In other words, the depth of 0–10 mm may be the only effective range of CPSM penetration.

## 3. Conclusions

- Various experiments, such as water permeability, rapid chloride permeability test (RCPT), mercury intrusion porosimetry (MIP), X-ray diffraction (XRD), scanning electron microscope (SEM), Fourier transform infrared spectroscopy (FT-IR), and thermogravimetric analyzer (TGA) were used to investigate the water-resisting capability of concrete crystalline penetration sealer materials (CPSM) to give assurance of the validity of the result. The evidence from the experiments can be interpreted as consistently confirming that the material is waterproof.
- The waterproof crystalline mechanisms of CPSM were observed. The SEM indicates that the acicular-structured crystals filling capillary pores for mortar substrate of the internal microstructure beneath the concrete surface were observed. The XRD and the FT-IR show that the main hydration products of CPSM are C-S-H gel and CaCO_3_. In addition, the MIP shows the CPSM with the ability to clog capillary pores of mortar substrate. Thus it reduces porosity and appears more effective in sealing pores or cracks. The formation of white compounds, mainly comprising CaCO_3_, Na_2_Si_3_O_5_·5H_2_O, and SiO_2_ from the XRD analyses, or a hydrate of No. 50 sieve of CPSM, a key component to explore its formation of acicular-shaped crystals, is the major crystalline formation of CPSM.
- Depth of penetration capability is within 0–10 mm of the sealer layer beneath the concrete surface, identified by the TGA result.

## Figures and Tables

**Table 1.** Concrete mix of test specimens (unit: kg/m^3^).

W/C ratio	Cement	Water	Sand	Gravel
0.65	323	210	717	1032

**Table 2.** Weight loss *vs.* temperature at various depths.

Depth (mm)	Weight loss (%)			
Temperature	0–5	5–10	10–15	15–20
105–440 °C	0.912	0.718	0.604	0.631
440–580 °C	1.659	1.002	0.715	0.695
580–995 °C	0.098	0.093	0.047	0.045
